# Population structure of guppies in north-eastern Venezuela, the area of putative incipient speciation

**DOI:** 10.1186/1471-2148-14-28

**Published:** 2014-02-17

**Authors:** Magdalena Herdegen, Heather J Alexander, Wiesław Babik, Jesús Mavárez, Felix Breden, Jacek Radwan

**Affiliations:** 1 Institute of Environmental Sciences, Jagiellonian University, Gronostajowa 7, Krakow 30-387, Poland; 2 Bamfield Marine Sciences Centre, Bamfield, British Columbia V0R 1B0, Canada; 3 Department of Biology, University of Victoria, Victoria, British Columbia V8W 2Y2, Canada; 4 Centro de Ecología, Instituto Venezolano de Investigaciones Científicas, Apartado 20632, Caracas 1020-A, Venezuela; 5 Laboratoire d’Ecologie Alpine, UMR 5553 CNRS-Université Joseph Fourier, BP 53 2233 Rue de la Piscine, Grenoble 38041, France; 6 Department of Biological Sciences, Simon Fraser University, Burnaby, British Columbia V5A 1S6, Canada; 7 Institute of Environmental Biology, Faculty of Biology, Adam Mickiewicz University, Umultowska 89, Poznan 61-614, Poland

**Keywords:** Gene flow, Introgression, Reproductive barriers, Sexual selection, mtDNA, Microsatellites

## Abstract

**Background:**

Geographic barriers to gene flow and divergence among populations in sexual traits are two important causes of genetic isolation which may lead to speciation. Genetic isolation may be facilitated if these two mechanisms act synergistically. The guppy from the Cumaná region (within the Cariaco drainage) of eastern Venezuela has been previously described as a case of incipient speciation driven by sexual selection, significantly differentiated in sexual colouration and body shape from the common guppy, *Poecilia reticulata*. The latter occurs widely in northern Venezuela, including the south-eastern side of Cordillera de la Costa, where it inhabits streams belonging to the San Juan drainage. Here, we present molecular and morphological analyses of differentiation among guppy populations in the Cariaco and San Juan drainages. Our analyses are based on a 953 bp long mtDNA fragment, a set of 15 microsatellites (519 fish from 20 populations), and four phenotypic traits.

**Results:**

Both microsatellite and mtDNA data showed that guppies inhabiting the two drainages are characterised by a significant genetic differentiation, but a higher proportion of the genetic variance was distributed among populations within regions. Most guppies in the Cariaco drainage had mtDNA from a distinct lineage, but we also found evidence for widespread introgression of mtDNA from the San Juan drainage into the Cariaco drainage. Phenotypically, populations in the two regions differed significantly only in the number of black crescents. Phenotypic clustering did not support existence of two distinct groupings, but indicated a degree of distinctiveness of Central Cumaná (CC) population. However, CC population showed little differentiation at the neutral markers from the proximate populations within the Cariaco drainage.

**Conclusions:**

Our findings are consistent with only partial genetic isolation between the two geographic regions and indicate that the geographic barrier of Cordillera de la Costa has not played an important role in strengthening the incomplete pre-zygotic reproductive barrier between Cumaná and common guppy. Significant phenotypic differentiation between genetically similar (in terms of neutral variation) populations suggests that mate choice can maintain divergence at sexually selected traits despite gene flow. However, neither genetic nor phenotypic clustering supported delineation of two species within the region.

## Background

Local ecological adaptations, sexual selection and genetic drift in isolated populations may lead to the emergence of reproductive isolation – a fundamental requirement for speciation [1-4]. However, speciation constitutes the extreme of the process of divergence, which does not necessarily lead to the birth of new species. The development of reproductive isolation is usually a gradual process, and diverging populations may spend millions of years exchanging genes before reproductive isolation, and thus speciation, is completed [2,5,6]. Speciation may be more likely if processes underlying reproductive isolation act in concert. For example, sexual selection may lead to divergent female preferences for male sexual ornaments, which may lead to pre-zygotic reproductive isolation and speciation [reviewed in [7,8]. However, evolution of such divergent preferences is more likely if gene flow between populations is limited [2,7,8]. Geographic isolation, either by distance or some kind of barrier to dispersal, is considered the most important factor facilitating isolation of gene pools and, in consequence, speciation [2].

Due to a relatively high degree of isolation, streams and rivers favour formation of highly structured populations of aquatic organisms [e.g. [9,10], which should facilitate speciation by limiting gene flow. Indeed, freshwater fish undergo speciation relatively more easily compared to marine fish [11]. However, some degree of gene flow between populations in different rivers is possible if they inhabit the same river drainage or, to a lesser extent, if migration between rivers in different drainages occasionally occurs, e.g. during floods, or via the sea if river mouths are close enough and species can survive increased salinity. Riverine environments therefore provide a good system for understanding the various factors that promote and constrain speciation.

A possible case of incipient speciation between fish populations occupying different drainages has been proposed for guppies (*Poecilia*) inhabiting streams of north-eastern Venezuela [12,13]. The common guppy (*Poecilia reticulata*) is an important model organism for the study of sexual selection and natural selection in the wild [14-18]. Recently the guppy has also been used as a model for studying speciation by divergent sexual selection [12,19]. *P. reticulata* was first collected from Guaire River in Caracas and described by Peters (1859). Another form of the guppy, which differs in male colouration and body shape from the common guppy, was first collected by Franklyn Bond in 1937, and later by John Endler in 1975, in the coastal town of Cumaná, in north-eastern Venezuela, and has been known as ‘Endler’s Livebearer’. Alexander and Breden [12] carried out the first detailed morphological analysis of the guppy from the Cumaná region. They found that several populations located within the city of Cumaná differed significantly in sexually selected traits and body shape from other guppies across the natural range. They called this form the Cumaná guppy and provided evidence for its partial pre-zygotic isolation from the common guppy. When allowed to interact simultaneously with males from a Cumaná population and from a common guppy population from the San Juan drainage, Cumaná females gave birth to 80% of offspring sired by Cumaná males, whereas among common guppy females the proportion of offspring sired by Cumaná males was only 30.5%. In a separate series of crosses the authors found no evidence for genetic incompatibility between the two populations (females from all crosses produced viable offspring, which reproduced successfully in backcrosses with parental types). This suggests that the difference in paternity reflects female preferences for conspecifics, as was also shown in earlier work [20]. Alexander and Breden [12] thus concluded that Cumaná guppies and common guppies may be undergoing speciation, with reproductive isolation arising as a consequence of divergent sexual preferences. However, it is not clear to what extent this partial reproductive isolation can limit gene flow between natural populations of Cumaná guppies and common guppies occurring in north-eastern Venezuela.

**Figure 1:**
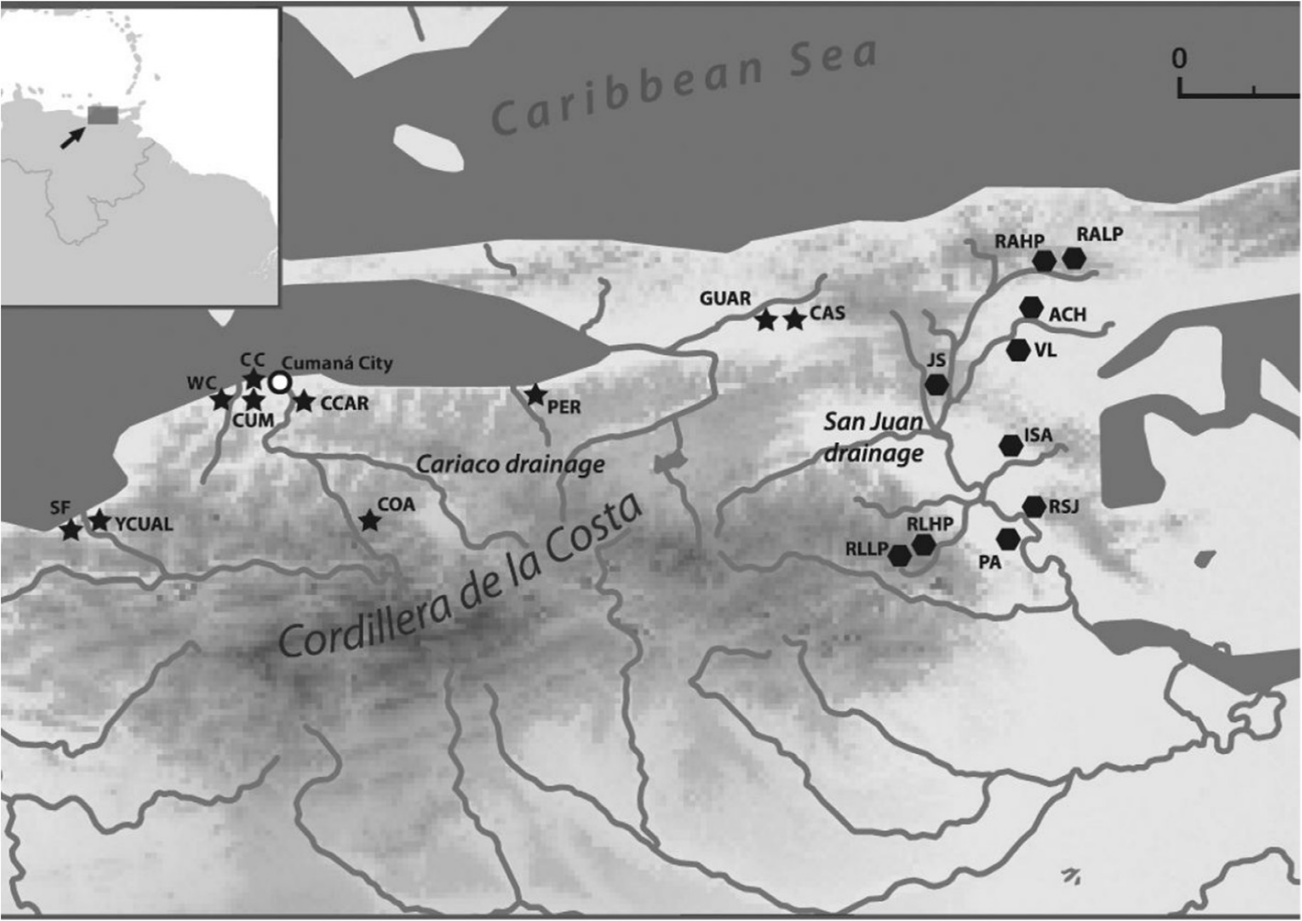
**Sampling sites in northern Venezuela.** Locations within the Cariaco drainage are marked with stars, within the San Juan drainage locations with hexagons; abbreviations of population names as in Additional file 1: Table S1.

Poeser *et al*. [13] argued that because of the differences in colouration, body and gonopodium shape and sexual behaviour, the Cumaná guppies, along with other populations inhabiting streams and rivers north-west of Cordillera de la Costa and draining to the Gulf of Cariaco and Caribbean Sea (Figure 1, henceforth referred to as Cariaco drainage populations), probably constitute a separate species, which they named *Poecilia wingei*. They proposed that the uplifting of Cordillera de la Costa, about 600,000 years ago, separated two groups of guppy populations, allowing *P. wingei* to diverge in allopatry from the common guppy populations inhabiting rivers of the San Juan drainage. According to this scenario, divergence in morphology and female preferences evolved under gene flow limited by the geographic barrier. However, Poeser *et al.*[13] provided no quantitative morphological, behavioural or genetic assessment of the differences between guppy populations in these two drainages.

Molecular evidence for genetic isolation between these groups of populations has so far been very limited and does not give a clear idea about gene flow between the two drainages. A study of mitochondrial DNA (mtDNA) control region sequences carried out by Alexander *et al.*[21] found that mtDNA haplotypes of Cumaná region form two distinct lineages, one of which includes haplotypes from the San Juan drainage. Even if this pattern suggests that there has been some introgression of mtDNA between Cariaco and San Juan populations, these populations may still be differentiated at nuclear genes, as mtDNA tends to be more sensitive to introgression than nuclear DNA, due to various phenomena, such as lower effective population sizes and different mobility of sexes [22-24]. A recent study [25] of genome-wide single nucleotide polymorphisms included the Cumaná population and one population from the San Juan drainage (Poza Azufre). The study showed high divergence between these two populations, but it remains to be seen to what extent the two populations reflect divergence between the two regions in general.

The main goal of this study was to expand the analyses of Alexander *et al*. [21], and to quantify genetic differentiation among populations in the north-western Cariaco drainage and south-eastern San Juan drainage, east of the Cordillera de la Costa. Two types of molecular markers were used: mtDNA and microsatellites. We also analysed phenotypic differentiation among populations in these drainages, using male body shape and sexually selected colour patterns which Alexander and Breden [12] found to differentiate the Cumaná guppy from the common guppy. If the Cordillera de la Costa is a major geographic factor facilitating incipient speciation in the guppy, one would expect significant morphological and genetic differentiation between the two drainages. In particular, we expected that significant genetic differentiation between the drainages coincides with significant phenotypic differentiation. Furthermore, we used Discriminant Analysis of Principal Components (DAPC) to investigate whether separating guppies in the region into two distinct species is justified by morphological differentiation.

## Results

### Microsatellites

Microsatellite loci were genotyped in 519 individuals. All 15 loci were polymorphic, with the number of alleles ranging from 14 to 93 (mean = 43.9). The genotyping error was estimated at 4.4%. Seventy-three of 1879 pairwise tests for linkage disequilibrium (3.9%) were significant after applying the sequential Bonferroni correction. However, there was no repeatable pattern of linkage disequilibria across populations; linkage disequilibrium for a given pair of loci was almost always detected in a single population only. This indicates that linkage disequilibria resulted from population structure/admixture rather than from physical linkage between loci. Significant departures of the genotype frequencies from Hardy-Weinberg expectations were detected, after correcting for multiple comparisons, in 57 out of 295 tests (19.3%, Additional file 1: Table S4). This was most probably due to the presence of null alleles, which were detected in all populations in at least some loci. Their estimated frequencies ranged from 0 to 0.36, but differed greatly for the individual loci between populations (Additional file 1: Table S5). Therefore, we did not exclude them from further analyses.

Comparisons between pairs of populations revealed a degree of genetic differentiation (*F*_ST_) ranging from 0.008 (between two adjacent *P. reticulata* populations) to 0.35 (between a pair of populations in different drainages), with global *F*_ST_ *=* 0.20. Most of the pairwise tests produced values between 0.1 and 0.3 (Additional file 1: Table S6). Multidimensional scaling of genetic distances showed that Cariaco and San Juan drainage populations cluster separately with the only exception of the population CAS, which clustered together with San Juan populations (Additional file 2: Figure S1). A similar pattern emerged from a Neighbour Joining tree constructed from the matrix of pairwise *F*_ST_ (Additional file 3: Figure S2), but separation between Cariaco populations (excluding CAS) and San Juan did not receive significant bootstrap support (0.46; see Additional file 3: Figure S2). A weak pattern of isolation by distance was detected when all populations were considered together (r = 0.14, P = 0.051, Mantel test, Additional file 4: Figure S3).

**Figure 2:**
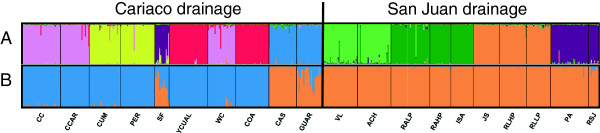
**STRUCTURE analysis based on 15 microsatellite loci.** Results for **A)** K = 8, **B)** K = 2. Cariaco drainage populations are to the left, San Juan to the right of the vertical line.

**Figure 3:**
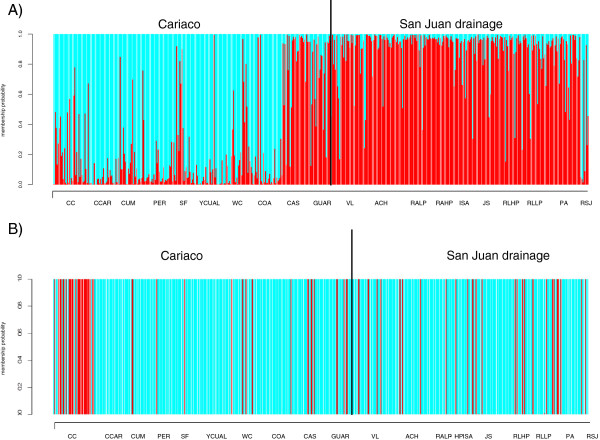
**Discriminant Analysis of Principal Components (DAPC) based on A) 15 microsatellite loci, B) four morphometric traits.** Results for the number of clusters K = 2.

The most likely number of genetic clusters identified in STRUCTURE analysis was 8. Generally, populations from Cariaco and San Juan drainages fall into separate clusters (Figure 2A), with the exception of SF, a Cariaco population which clustered together with two San Juan populations, PA and RSJ. We also present the result of STRUCTURE clustering for K = 2 (Figure 2B) which was performed to check whether populations on each side of the geographic barrier (Cordillera de la Costa) form separate clusters when two genetic clusters are assumed. In this analysis, populations from the Cariaco drainage and San Juan drainage grouped separately, except for two anomalies; the SF population, which contains an admixture of alleles from San Juan drainage, and the CAS and GUAR populations, which group with San Juan drainage populations. In the DAPC fifteen principal components of PCA and three discriminant functions were retained. As in STRUCTURE, K = 8 appeared optimal. As expected, DAPC yielded similar results to STRUCTURE for both K = 8, (not shown) and K = 2 (Figure 3A). The mean probabilities of assignment of the individuals to the cluster representing their own drainage were: 0.73 (SD = 0.33, SE = 0.04) and 0.86 (SD = 0.21, SE = 0.05), for Cariaco and San Juan drainages, respectively. The mean probability of assigning San Juan drainage guppies to the cluster representing Cariaco drainage was 0.14 (SD = 0.20, SE = 0.01). Analysis of variance showed a highly significant difference in assignment probabilities of individuals differing in their drainage of origin into cluster 1 representing the Cariaco drainage (F_1.18_ = 57.12, P = 0.000).

AMOVA analyses with groups defined on the basis of mtDNA (i.e. lineages A and B vs. lineage C, see below), indicate that 4.17% of the total microsatellite variation was explained by the group level (P < 0.001). Groups that were geographically defined (Cariaco vs. San Juan drainage) explained 7.83% of variation when all populations were included (2001 and 2011 samples), and 5.93% when only the populations sampled in 2011 were included (P < 0.001 in both cases).

### Mitochondrial DNA

**Figure 4:**
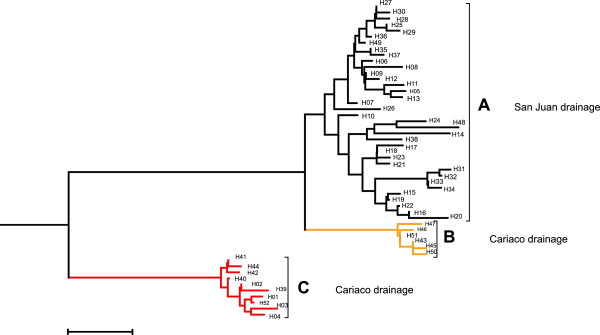
**Mitochondrial neighbour-joining tree.** The tree shows the relationships between 52 *P. reticulata* haplotypes; **A)** haplotypes of guppies from San Juan drainage (with few exceptions; see Additional file 1: Table S1), **B** and **C)** haplotypes of guppies from Cariaco drainage; *P. latipinna* was used as the outgroup.

Among 106 sequenced individuals, 52 haplotypes were identified [GenBank Acc. No. KJ415678-KJ415729]. Most mtDNA haplotypes clustered into two major well-supported lineages (Figure 4), one of which occurs exclusively among Cariaco drainage guppies (populations CC, CCAR, CUM, PER and SF, lineage C in Figure 4, Additional file 5: Figure S5). Inside the second lineage a well-supported sub-lineage is distinguishable, occurring exclusively among Cariaco drainage guppies (lineage B: populations WC, COA, YACUAL and CC; Figure 4, Additional file 1: Table S7, Additional file 5: Figure S5). The remaining haplotypes clustering with the sub-lineage B (which we call lineage A for convenience) occurred predominantly among the San Juan drainage guppies, except for a few haplotypes representing CAS (H9, H12 and H24), GUAR (H48 and H49) and SF (H38) populations (Additional file 1: Table S7, Additional file 5: Figure S5). The RFLP analysis confirmed that the haplotypes common in lineage C were predominant in CC, CCAR, CUM, PER and SF populations (Additional file 1: Table S7, Additional file 5: Figure S5). The net divergence between the two main mtDNA lineages (C vs. A, B) was 0.035.

Pairwise *F*_ST_ between populations for mtDNA ranged from 0 to 1, and 97 tests were significant (Additional file 1: Table S8). Drainage explained 28.72% of mtDNA differentiation (AMOVA; all populations grouped by drainage), a considerably higher proportion of the variance than in the case of microsatellites. This percentage increased to 55.52%, when the six populations sampled in 2001 were excluded (P < 0.001).

### Colour and shape variation

**Figure 5:**
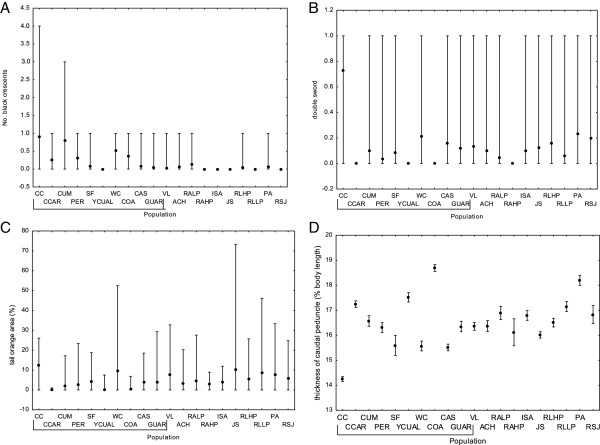
**Phenotypic traits of the sampled populations. (A)** mean number of black crescents, **(B)** mean number of double swords, **(C)** mean relative orange area on the caudal fin, **(D)** mean relative thickness of caudal peduncle; error bars represent minimum and maximum values for **A**, **B** and **C**, and standard error for **D**. Cariaco drainage populations are underlined.

Populations differed significantly in all three colouration characteristics (black crescents, *χ*^2^ = 127.75, P < 0.001; double swords, *χ*^2^ = 117.23, P < 0.001 and tail orange area, *χ*^2^ = 164.23, P < 0.001; Kruskal-Wallis test; Figure 5A-C). The mean number of black crescents differed significantly between Cariaco and San Juan guppies (P = 0.006, Mann–Whitney test). The presence of double swords and relative tail orange area did not differ significantly between the two drainages (P = 0.518 and P = 0.307, respectively; Mann–Whitney test). The relative thickness of the caudal peduncle did not differ between drainages (F_1,18_ = 0.49, P = 0.490) but was significantly differentiated among populations (F_18,422_ = 32.43, P < 0.001, Figure 5D).

In DAPC for phenotypic traits five principal components of PCA and three discriminant functions were retained. We found no support for the presence of two phenotypically distinct clusters which could be equated with two species. Indeed, the values of BIC did not reach a plateau, providing no clear indication that the optimal number of clusters was less than 20 (Additional file 6: Figure S4). Therefore, we only performed the analysis for two clusters (K = 2) to test the main hypothesis about differentiation between drainages (Figure 3B). The mean probabilities of assignment to the cluster representing own drainage were: 0.16 (SD = 0.36, SE = 0.01) and 0.87 (SD = 0.34, SE = 0.06), for Cariaco and San Juan drainages, respectively. The mean probability of assigning San Juan drainage guppies to the cluster representing Cariaco drainage was 0.13 (SD = 0.34, SE = 0.01). There was no difference in assignment probabilities of individuals differing in their drainage of origin into cluster 1 representing the Cariaco drainage (*χ*^2^ = 95.94, P = 0.49).

## Discussion

Cumaná guppy inhabiting a stream within the Cariaco drainage has been shown to be distinct in terms of male sexual colouration, and partially reproductively isolated form a common guppy population inhabiting a stream in the San Juan drainage [12]. The two drainages in north-eastern Venezuela are adjacent to each other, but are separated by a mountain range, Cordillera de la Costa, which may have formed a barrier to gene flow. It has been hypothesised that this barrier may have facilitated the phenotypic differentiation between guppy populations inhabiting separated drainages [13]. To test this hypothesis, we studied guppy populations inhabiting these two drainages using both molecular (mtDNA and microsatellites) and morphometric approaches.

In accordance with the hypothesis that the Cordillera de la Costa constitutes a barrier to gene flow, the grouping of populations by drainage explained a significant proportion of genetic variation in both mtDNA and microsatellites. STRUCTURE results generally supported the distinction between the Cariaco and San Juan drainage guppies. In the analysis with two genetic clusters imposed, the populations clustered mostly by drainage (see Figure 2B), the exception being the populations CAS, GUAR and SF, located near the western and eastern borders of the Cariaco drainage. DAPC yielded similar results, with GUAR and CAS showing the greatest admixture of genes from the San Juan cluster, and all other populations clustering in accordance with drainage membership (see Figure 3A). Multidimensional scaling of the matrix of pairwise *F*_ST_ showed a similar pattern of separation between drainages, except one Cariaco drainage population (CAS) which is separated from San Juan drainage cluster along the first dimension (see Additional file 2: Figure S1).

Microsatellite differentiation between the Cariaco and San Juan drainages (7.8% for all populations sampled, and 5.93% for populations sampled in 2011) was larger than that reported by Suk and Neff [26] for differentiation between guppies in the Caroni and Oropuche drainages in Trinidad (4.6%), a level of differentiation proposed by Schories *et al.*[27] to represent separate *Poecilia* species. For comparison, in the smooth (*Lissotriton vulgaris*) and Carpathian (*L. montandoni*) newts which constitute well defined species, 7.79% microsatellite variation was attributed to interspecific differentiation [28], a value comparable to the one reported here. However, different races of the genus *Heliconius,* which are considered examples of incipient speciation, were found to differ by over 30% at microsatellite loci [29]. These examples imply considerable variation in the degree of differentiation at microsatellite loci among incipient/young species. However, microsatellites generally have not supported clustering together of populations according to the region (see Additional file 3: Figure S2), providing no evidence of species status to populations from the Cariaco region.

In the mtDNA phylogeny, two main lineages were distinguished; one composed exclusively of haplotypes from the Cariaco drainage, the other comprising mostly haplotypes found in guppies from the San Juan drainage, but containing also a well-supported Cariaco sub-lineage B. Both the Cariaco drainage lineage C and sub-lineage B received a high bootstrap support, indicating its distinctiveness and substantial divergence from the San Juan lineage (Figure 4), as reported by Alexander *et al.*[21] for the mtDNA control region. Assuming mutation rate typical for many vertebrates (divergence of 2% per million years; [5]), the net divergence between the two main lineages of haplotypes (0.035) would be consistent with the 600,000 years of separation between the two drainages starting with the uplifting of Cordillera de la Costa, although this is only a rough estimate and should be treated with caution.

While mtDNA and microsatellite divergence estimates reported above support the hypothesis of considerable isolation and differentiation between guppies inhabiting streams on the opposite sides of the Cordillera de la Costa, STRUCTURE results do not corroborate such a clear distinction. The Evanno *et al.*[30] method indicated eight, not two, as the most probable number of genetic clusters. While this result should be treated with caution, as STRUCTURE is not well suited for the analysis of data exhibiting hierarchical population structure, DAPC yielded the same optimal number of clusters. Furthermore, a greater number of clusters than two is consistent with the results of AMOVA, which showed that most genetic variation is distributed between populations within drainages. The fact that the highest degree of admixture was observed in populations near the borders of the drainages (CAS, GUAR, SF, see Additional file 5: Figure S5) strongly indicates that this pattern is due to gene flow rather than homoplasy. Gene flow is further corroborated by mtDNA introgression, which shows similar geographic pattern.

Neighbouring populations and populations sampled from within the same stream mostly fall into the same cluster (e.g. CAS and GUAR, both sampled from Rio Casanay, and VL and ACH or PA and RSJ, population pairs sampled from adjacent streams). Also populations CC, CCAR and WC, which are located in the Cumaná flood plain, were assigned to a common cluster. This pattern is reflected in significant, although weak, isolation by distance.

Interestingly, the population CUM, (ca. 1 km distance from CC which was sampled in 2011), clusters with a more distant population PER, which was sampled during the same season as CUM, 10 years after the first three populations were sampled. This genetic similarity between geographically more distant populations, but temporally closer sampling events indicates that temporal changes in genetic composition of populations may be rapid and have a considerable effect on population structure of guppies in north-eastern Venezuela. Recent simulations [31] showed that large temporal allele frequency fluctuations can arise as a consequence of relatively small sizes of guppy populations [32]. Despite this, drainage explained a similar percentage of variation in microsatellites when samples from 2001 were excluded, and for mtDNA the structure was even more pronounced in this limited dataset. Therefore, the overall picture of significant differentiation between drainages was not biased due to a temporal gap in sampling events from the Cariaco drainage.

Surprisingly, SF located at the western border of the region did not fall into any group composed of Cariaco drainage populations in the STRUCTURE analysis, and instead shared the cluster with the geographically distant San Juan drainage populations. A possible explanation could be that introgression from a western common guppy population occurred; indeed, individuals with the haplotype from haplogroup A, typical of San Juan drainage guppies, were found in populations located west of SF (MH and JR, unpublished data).

Our results suggest a recent increase in introgression of San Juan genes into Cariaco drainage guppies (please note, that we are using the term introgression to indicate gene flow between genetically differentiated groups within a species; we do not suggest that we are dealing with two separate species). Two pairs of populations in our data, sampled from very close locations, YCUAL/SF and GUAR/CAS (in each pair sampled in 2001 and 2011, respectively), corroborate the idea of rapid invasion happening in few years. In the STRUCTURE analysis with two main genetic clusters, YCUAL showed more genetic similarity to guppies in the Cariaco drainage, while SF sampled 10 years later in close proximity showed a high proportion of the San Juan genes. In the second case, population GUAR, sampled in 2001, clustered to a great extent with San Juan populations but also showed considerable admixture with Cariaco genes, whereas CAS, sampled in 2011 from the same stream, grouped with the San Juan drainage populations. A similar situation has been reported for Trinidadian guppies, where substantial admixture was found between populations in adjacent streams, but belonging to different drainages ([26]; based on 7 microsatellite loci). Furthermore, in populations at the borders of the Cariaco and San Juan drainages, CAS and GUAR, only mitochondrial haplotypes typical for San Juan drainage were present, and one was also found in SF (see Additional file 1: Table S7, Additional file 5: Figure S5). This clear geographical pattern strongly suggests introgression, rather than incomplete lineage sorting, as an underlying mechanism. A recent SNP-based study also indicated introgression from Poza Azufre (SanJuan drainage) to Cumaná (see Figure 3 in [25]).

Our data also provide evidence that some introgression may have occurred in a more distant past. In a phylogram of mitochondrial haplotypes within the San Juan lineage (Figure 4), a Cariaco sub-lineage was clearly distinguished, supported by a high bootstrap value. The presence of this lineage may be explained by a past introgression of a San Juan haplotype into Cariaco populations, which then evolved, in isolation from San Juan guppies, within these populations.

The reasons for the San Juan drainage guppy’s invasion into the Cariaco drainage are not clear. The pattern of introgression, most pronounced near the borders of the Cariaco drainage, suggests the role of natural processes and not of human-induced introductions. We are not aware of any abrupt environmental changes in this area, but occasional floods occurring in the region may favour occasional migration events [26]. AMOVA results based on microsatellite loci indicate the proportion of variation explained by drainage was nearly twice as high as that estimated in the analysis in which the highest level of hierarchy was defined by mtDNA haplotypes. This suggests that when the geographic barrier is crossed, reproductive barriers do not prevent introgression of mtDNA.

The analysis of body shape and colour patterns that distinguish the Cumaná guppy from other guppy populations [12] showed that only the population CC represented all the characteristics which phenotypically define the Cumaná male morphotype (presence of double sword, presence of black crescents, larger tail orange area and narrower caudal peduncle, relative to typical *P. reticulata*). Some other populations from Cariaco drainage exhibited individual Cumaná characteristics, but not their complete combination (see Figures 3B and 5). Individuals from the WC and CUM populations, located near the CC population, had an increased number of black crescents, while the WC population revealed an increase in the mean orange tail area and, together with CAS, a relatively high occurrence of the double sword, compared to other guppy populations measured. The only trait which significantly differed between the Cariaco and San Juan drainages was the presence of black crescents. Thus overall, there is little evidence that most guppies inhabiting Cariaco region can be equated with Cumaná morphotype, as suggested by Poeser [13]. Indeed, most individuals from CC population were assigned to cluster 1 in the DAPC based on phenotypic traits, whereas vast majority of individuals from other populations from both San Juan and Cariaco drainages, were assigned to cluster 2 (see Figure 3B). Consequently, there was no significant difference between drainages in probability of assignment to a drainage. This result clearly shows that morphological differences are not aligned with a geographic barrier between two drainages. Rather, the CC population represents the typical Cumaná morphotype, with other traits, except for black crescents, occurring only in some populations in the region.

That we found no evidence for original designation of *P. wingei* according to drainage [13] does not exclude the possibility that Cumaná population could be genetically and morphologically distinct enough to be considered a different species. However, our results do not provide evidence for genetic differentiation, as CC population, representing Cumaná morphotype, clustered together in the STRUCTURE with CCAR which represents the common guppy morph (see Figures 2A and 5). Furthermore, phenotypic analyses showed that populations in the San Juan drainage also contained individuals with some ‘Cumaná traits’, for example, double swords were observed in the Poza Azufre and Rio San Juan populations, and high mean orange area in Rio Juan Sanchez population. This pattern is visible on the graphical representation of DAPC, where individuals belonging to the cluster 1, dominated by the ‘Cumaná-type’ phenotypic characteristics, occur sporadically throughout all populations (see Figure 3B). Likewise, CC population contained individuals assigned to 'common guppy' cluster 2 (Figure 3B). Overall, our data do not support existence of two species which can be easily separated based on male morphology. The lack of support for the existence of two clusters in DAPC analysis of morphological traits further strengthens this conclusion.

There are two possible explanations for the patterns we observed. First, the Cumaná city area constituted the source of Cumaná morphotype, and migrants from this area spread widely throughout the Cariaco drainage. The second is that the whole Cariaco drainage was the original range of the Cumaná type guppy, but the populations located closer to the border with the San Juan guppies have been invaded by immigrants from the San Juan drainage.

As most genetic variance was distributed among populations rather than between drainages, it seems that limited gene flow between different streams, to a greater degree than between drainages, may have facilitated the evolution of divergent female preferences of females form Cumaná [12]. Apart from limited gene flow, genetic divergence between populations may be due to drift, as effective sizes of many guppy populations are <1000 [32]. A recent theoretical model showed that under such conditions, speciation by sexual selection is particularly likely [33].

Once divergent mating preferences evolve, they may constitute a powerful mechanism maintaining divergence in sexual traits. Genetic clustering of populations from the Cumaná flood plain (Figure 2A), representing both Cumaná (CC population) and common guppy morphs (CCAR) suggests that female choice has imposed differential rates of introgression among genes that do or do not encode for traits related to biological divergence. Divergent female preferences have also been shown to facilitate reproductive isolation among guppy populations differing in the strength of selection from predators [34]. Thus, although sexual selection can under some scenarios also constrain speciation [35], our results are consistent with a traditional view that sexual selection may facilitate the emergence of reproductive isolation [2]. However, we have also found that phenotypic traits typical for Cumaná morphotype are present in other populations and vice versa, which indicates that the barrier to the flow of genes associated with traits subject to mating preferences is incomplete. More intensive sampling effort in Cumaná region will be necessary to determine if morphological homogenisation with respect to male sexual traits has been occurring in consequence.

## Conclusions

Our results show significant genetic differentiation between guppy populations in neighbouring drainages, but the geographic barrier of Cordillera de la Costa played a minor role in structuring guppy populations relative to the influence of limited gene flow between streams within drainages. Our results indicate that immigration of San Juan drainage guppies into the Cariaco drainage has been occurring both in the past and recently, and the resulting introgression may be causing erosion of genetic differentiation of the guppies it the Cariaco region. Phenotypic differentiation between regions did not correspond to genetic differentiation, thus providing no evidence for the role of geographic barrier in incipient speciation. Instead, our analyses supported phenotypic distinctiveness of males from Central Cumaná population. Significant phenotypic differentiation between genetically homogenous (in terms of neutral variation) populations from Cumaná region suggests that divergent mate preferences documented in earlier work [12] can maintain variation in secondary sexual traits even in the face of considerable gene flow. However, most phenotypic traits characterising Cumaná morphotype were not unique to this population, resulting in assignment of many individuals from other populations to Cumaná phenotypic cluster and vice versa, indicating that pre-zygotic barrier to the flow of genes associated with divergent male colouration is incomplete. Overall, our data do not provide justification for distinguishing the separate species *P. wingei*.

## Methods

### Samples

Male and female guppies from 20 populations in north-eastern Venezuela were collected in two separate trips (2001 and 2011). In total 519 individuals were used for this study. Samples from six populations (CC, CCAR, COA, WC, YCUAL and GUAR) were collected in 2001; a total of 173 fish with an average of 27 individual males per population. The remaining fourteen samples were collected in 2011; a total of 346 fish with an average of 25 individuals (males and females) per population. Fish were collected from streams, rivers or canals using a dip net, killed with an overdose (~0.03% solution) of the anaesthetic MS-222 (tricaine methanosulfonate), photographed and fixed for later molecular analyses in 95% ethanol. The names, sample sizes and geographic coordinates of sampling sites are given in Additional file 1: Table S1. Fish were collected in collaboration with D. Taphorn of Universidad Nacional Experimental de los Llanos Occidentales (permit no. 0497).

### Molecular methods

Total genomic DNA was extracted from tail fin or tail muscle from male fish collected in 2001 using standard phenol/chloroform isolation [36] or using the PURGENE DNA Isolation Kit (Gentra Systems, Inc., Minneapolis, MN, USA). Genomic DNA from male and female fish sampled in 2011 was extracted using the Wizard® Genomic DNA Purification Kit (Promega).

All (519) individuals were screened for allelic variation at fifteen previously described microsatellite loci (Additional file 1: Table S2): AG1 and AG9 [37], G75, G183, G211, G255 and G325 [38], Pret-52, Pret-48 [39] , Pr92 [40], TACA033 [GenBank Acc. No. AY258896], CA061 [GenBank Acc. No. 30 AY258683], TAGA033 [GenBank Acc. No. 258667], Pre15 [GenBank Acc. No. AY830943], Pre26, [GenBank Acc. No. AY830946].

Loci were amplified with Multiplex PCR Master Mix (Qiagen), in 5 multiplexes with one primer in each primer pair fluorescently labelled (Additional file 1: Table S2). The 10 μl PCR mixture included 5 μl of Master Mix, 0.2-0.4 μM of each primer (Additional file 1: Table S2) and 20-100 ng of genomic DNA. The reaction conditions were: a 15 min denaturation step at 95°C, 36 cycles of 30 s at 94°C, 1 min at 52°C followed by 1 min at 72°C, and 10 min of final extension at 72°C. PCR products were mixed with GeneScan 500 LIZ size standard and electrophoresed on an ABI 3130xl. Genotyping was performed in GeneMapper 4.0 (ABI). In order to estimate the genotyping error we repeated the procedures for 4% of samples.

A 953 bp fragment of mtDNA cytochrome b gene was amplified in 504 individuals, using primers designed on the basis of available mitochondrial sequences [GenBank Acc. No. GQ855708.1- GQ855739.1]. The 10 μl PCR mixture contained 5 μl of Hot Start PCR Master Mix (Fermentas), 1 μM of forward and 1 μM of reverse primer (Additional file 1: Table S3), and 10-50 ng of genomic DNA. The PCR conditions were as follows: a 4 min denaturation step at 95°C, followed by 35 cycles of 30 s at 95°C, 45 s at 55°C, 1 min at 72°C, and a final extension at 72°C for 10 min. For five to six individuals from each population (a total of 106 individuals) PCR products were sequenced using Big Dye Terminator v. 3.1 and products of sequencing reactions were separated on an ABI 3130xl. In addition to amplification primers, two internal primers were used for sequencing (Additional file 1: Table S3). We found that a group of haplotypes common in the Cariaco region, including some populations in the Cumaná city (lineage C in Figure 4) differed substantially from the remaining haplotypes (lineages A and B in Figure 4). Therefore we were able to identify restriction sites that distinguish the C haplogroup from the remaining two groups of haplotypes and we used Restriction Fragment Length Polymorphism (RFLP) analysis to assign almost all remaining samples to haplotype groups/lineages. We digested PCR products with restriction enzyme *Hpy188III*, which has two cutting sites in the amplified cytochrome *b* fragment in haplotypes from lineages A and B (corresponding to positions 191 and 738 in complete cytochrome *b* sequence; GenBank Acc. No. GQ855711.1), producing fragments of 113, 294 and 546 bp. In contrast, there are no cutting sites in the C lineage for that enzyme. Digestion products were electrophoresed on a 1.5% agarose gel containing GelRed.

### Population genetic analyses

#### Microsatellites

The presence of null alleles was tested using FreeNA [41]. In each population, loci were checked for Hardy-Weinberg equilibrium and linkage equilibrium using exact tests implemented in Genepop 4.1.2 [42]. A sequential Bonferroni correction was applied to control for multiple tests. Pairwise *F*_ST_ values between populations, adjusted for the presence of null alleles using the excluding null alleles (ENA) correction [41], were calculated in FreeNA, and visualised with multidimensional scaling performed in Statistica v.10 (Statsoft). Relationships among populations were also visualized as a Neighbour Joining tree constructed from the matrix of pairwise *F*_ST_ using POPTREE2 [43]. Robustness of the tree was tested with 1000 bootstrap replicates.

Analysis of molecular variance was performed in Arlequin 3.1 [44] to assess the proportion of microsatellite variation explained by various levels of hierarchical structure. Three separate analyses were performed which differed in the definition of the highest structure level (group) or in the dataset used. In the first analysis two groups of populations were distinguished based on mitochondrial differentiation, corresponding to lineages A and B vs. lineage C. The C lineage included populations CC, CCAR, CUM, PER and SF. In the second analysis two groups (Cariaco drainage and San Juan drainage) were defined based on the geographic criterion as proposed by Poeser *et al.*[13]. The group identified as ‘Cariaco drainage’ included all C-lineage populations but was extended to include YCUAL, WC, COA, CAS, and GUAR populations. The third analysis repeated the second but included only those populations sampled in 2011, to test whether temporal changes in the genetic composition of populations affected the variance explained by the drainage. Correlation between the genetic (linearised pairwise *F*_ST_ values) and linear log-geographic distance between populations was tested using Mantel’s test in IBDWS 3.23 [45].

To infer the most probable number of genetically differentiated clusters we analysed the microsatellite data from all populations in STRUCTURE 2.3.3 [46-48], using the admixture model with uncorrelated allele frequencies and assuming the presence of null alleles. Alpha value was set to 7 to achieve good mixing. The burn-in length was set to 100,000 and the post-burn-in MCMC was run for one million steps. Values of K (the number of genetic clusters) from 1 to 20 (the number of sampled populations) were tested, and for each K 10 runs were performed. Structure Harvester [49] was used to calculate ΔK, a measure that estimates the most probable number of clusters [30]. Additionally, analysis of principal components (DAPC, [50,51]) was performed on the same set of microsatellite data. The analysis was performed in R 3.0.1 [52], using the package “*adegenet*” [50]. This method yields similar information to STRUCTURE but, contrary to STRUCTURE, it is applicable not only to genetic data. This feature of DAPC enables to compare different variables, and we used this feature to compare genetic and morphological data (see below). We imposed the number of clusters (K) of two and hypothesized that the populations would then split following the pattern of the two drainages. Number of retained principal components was determined based on the graph of variance explained by PCA and discriminant functions based on the bar plot of eigenvalues for discriminant analysis, as recommended by Jombart et al. [51]. A hierarchical ANOVA (populations were nested in drainages) was performed on the probability values of assignment of each individual to cluster 1, containing CC population. The expectation was that individuals belonging to the drainage identified as cluster 1 will show a significantly higher probability of assignment to cluster 1 than will have the other individuals. Additionally, mean probability of assignment to each of the clusters was calculated separately for populations from both drainages.

### Mitochondrial DNA

Mitochondrial sequences were checked for quality and aligned in SeqScape 2.5 (ABI). Phylogeny was reconstructed in MEGA 5.05, using the neighbour-joining method and assuming Jukes-Cantor model; this simple model of sequence evolution corrects for multiple substitutions and is characterised by a lower variance compared to more complex models, which is desirable, particularly when the overall divergence is low, as in the present case [53]. Net sequence divergence between the two main lineages (A and B vs. C, see Results) was estimated in MEGA using the Jukes-Cantor model. This approach estimates divergence with correction for polymorphisms segregating within lineages and alleviates the effect of an apparent acceleration of the rate of recent sequence evolution due to segregation of deleterious polymorphisms [54]. Pairwise *F*_ST_ between populations and *F*_ST_ between Cariaco and San Juan drainages were calculated in Arlequin 3.1 [44]. To assess mtDNA differentiation at two levels of hierarchy between Cariaco and San Juan drainages and among populations within the regions, we run AMOVA in Arlequin 3.1 [44]. To examine whether there was an effect of sampling dates, two sets of AMOVA were performed: in the first all populations were included, whereas the second was performed using only populations sampled in 2011.

### Colour patterns and body shape

Phenotypic traits were measured on 442 males using ImageJ software [55]. These included colour traits and a body shape variable that differentiate the Cumaná guppy from the common guppy [12]: number of black crescents, presence/absence of a double sword on the caudal fin (sword-like orange patches on the dorsal and ventral margins), tail orange area (relative to total tail area), and the thickness of the caudal peduncle (relative to body length, tail fin excluded).

For the relative thickness of the caudal peduncle, a hierarchical ANOVA was performed with populations nested within regions. The distributions of residuals for black crescents, double swords and tail orange area were not normal (as assessed from normal-probability plots), and a large number of zero values precluded transformation. Hence, for these three traits non-parametric tests were used; the differentiation among populations was tested using a Kruskal-Wallis test, whereas comparison between drainages was analysed using a Mann–Whitney test performed on population means.

A discriminant analysis of principal components (DAPC) was performed for the morphometric variable composed of the four phenotypic traits (data for 422 individuals). Optimal number of clusters was assessed from the graph of BIC values versus number of clusters [51]. As for the molecular data, we also imposed the number of clusters (K) of two. As the assignment probabilities took 0 and 1 values (except three cases of probabilities >0.9 which we converted to 1), we used a generalized linear model with binomial error distribution (with populations nested in drainages) to test the expectation that individuals sampled from a given drainage show a significantly higher probability of assignment to that drainage than do individuals sampled from the other drainage. Additionally, mean probability of assignment to each of the clusters was calculated separately for populations from both drainages.

## Availability of supporting data

Supporting tables are figures are available as additional files. Mitochondrial haplotype sequences are deposited in GenBank [GenBank Acc. No. KJ415678-KJ415729].

## Competing interests

The authors declare that they have no competing interests.

## Authors’ contributions

MH took part in the study design, carried out molecular, statistical and population genetic analyses and drafted the manuscript. HA took part in the study design and molecular and statistical analyses, contributed photographs and tissue samples from Venezuela, and helped to draft the manuscript. WB supervised molecular and population-genetic analyses and helped to draft the manuscript. JM and FB took part in the study design and helped to draft the manuscript. JR took part in the study design, coordinated the study, supervised statistical analyses and helped to draft the manuscript. All authors read and approved the final manuscript.

## Supplementary Material

Additional file 1: Table S1 Names and abbreviations, sample sizes, sampling year and coordinates of the sampling locations. **Table S2.** List of primers used for microsatellite amplification. **Table S3.** Sequences of primers used to amplify (1,2) and to sequence (1-4) fragments of mitochondrial cytochrome b gene. **Table S4.** Hardy-Weinberg equilibrium tests for each locus in each population (Genepop); reported p values are not corrected; missing values are due to monomorphism of the locus in this particular population; p values significant after sequential Bonferroni correction are in red. **Table S5.** Null alleles frequencies for each locus in each population. **Table S6.** Pairwise *F*_ST_ values for microsatellites for all pairs of populations, values after ENA correction (FreeNA); Cariaco drainage populations in red, San Juan drainage populations in blue. **Table S7.** Distribution of mtDNA cytochrome b haplotypes among populations; numbers indicate number of individuals with a given haplotype; Cariaco drainage populations in red, San Juan drainage populations in blue. **Table S8.** Pairwise Fst values for cytochrome b for all pairs of populations, p = 0.05; significant values are followed by asterisk; Cariaco drainage populations in red, San Juan drainage populations in blue. Click here for file

Additional file 2: Figure S1 Multidimensional scaling. Two-dimensional scaling of the matrix of pairwise *F*_ST_ values between all sampled populations based on microsatellite frequencies; Cariaco drainage populations are enclosed in grey lines. Click here for file

Additional file 3: Figure S2 Neighbor Joining tree showing relationships among populations. The tree was constructed from the matrix of pairwise *F*_ST_ and its robustness was tested with 1000 bootstrap replicates. Cariaco drainage populations are in frames. Click here for file

Additional file 4: Figure S3 Isolation by distance. Relationship between genetic (*F*_ST_) and geographic distances between populations; r = 0.14, P = 0.051. Click here for file

Additional file 5: Figure S5. Geographic distribution of mitochondrial lineages. Colours correspond to the three main lineages: A – black, B – yellow, C – red. Click here for file

Additional file 6: Figure S4 Inference of the optimal number of genetic clusters. Graph output of the function *find.clusters* performed on morphometric data to identify the optimal number of clusters; Bayesian information criterion (BIC) on y axis. Click here for file
