# Supplementary figures and images for: Population structure of guppies in north-eastern Venezuela, the area of putative incipient speciation
Source: BMC Evol Biol. 2014 Feb 17;14:28. doi: 10.1186/1471-2148-14-28 (PMC3942120; doi:10.1186/1471-2148-14-28)

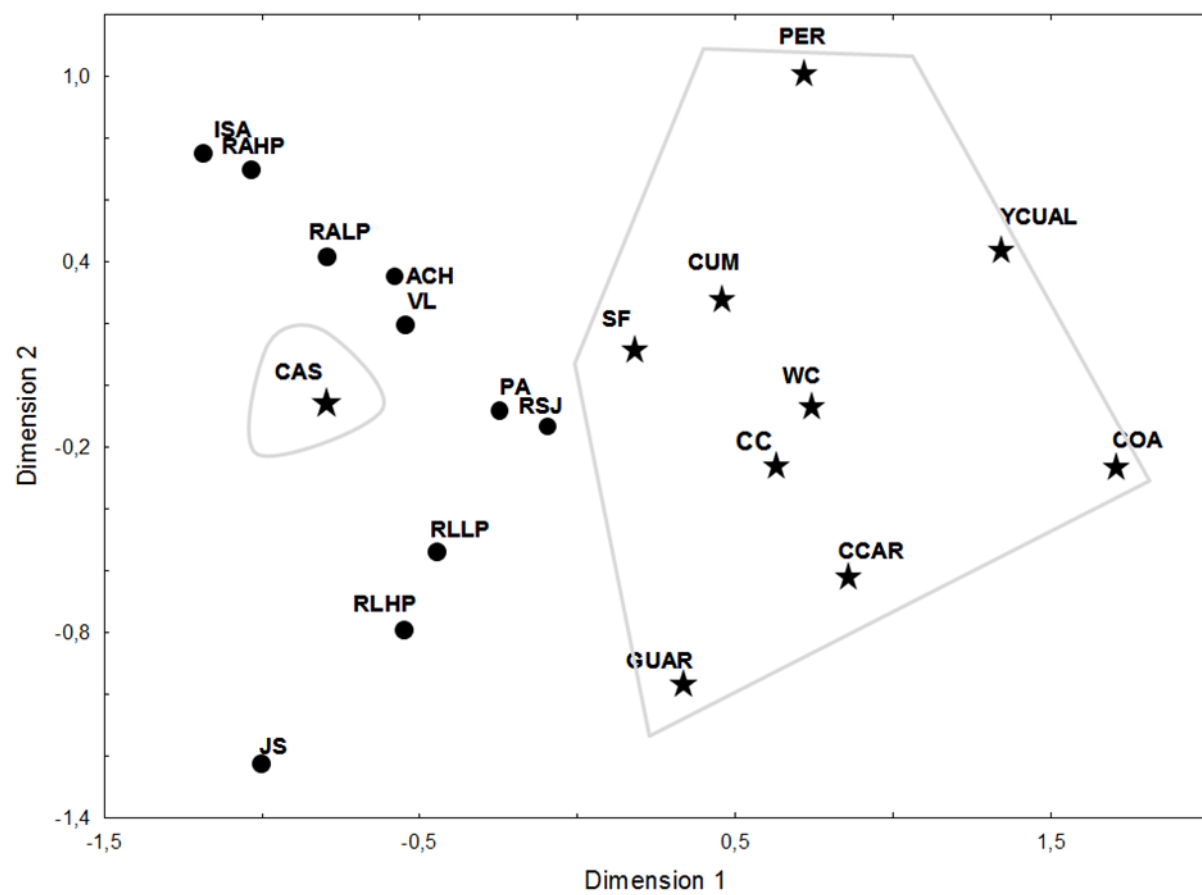

Supplement: Additional file 2: Figure S1 — Multidimensional scaling. Two-dimensional scaling of the matrix of pairwise FST values between all sampled populations based on microsatellite frequencies; Cariaco drainage populations are enclosed in grey lines. [file 1471-2148-14-28-S2.pdf]

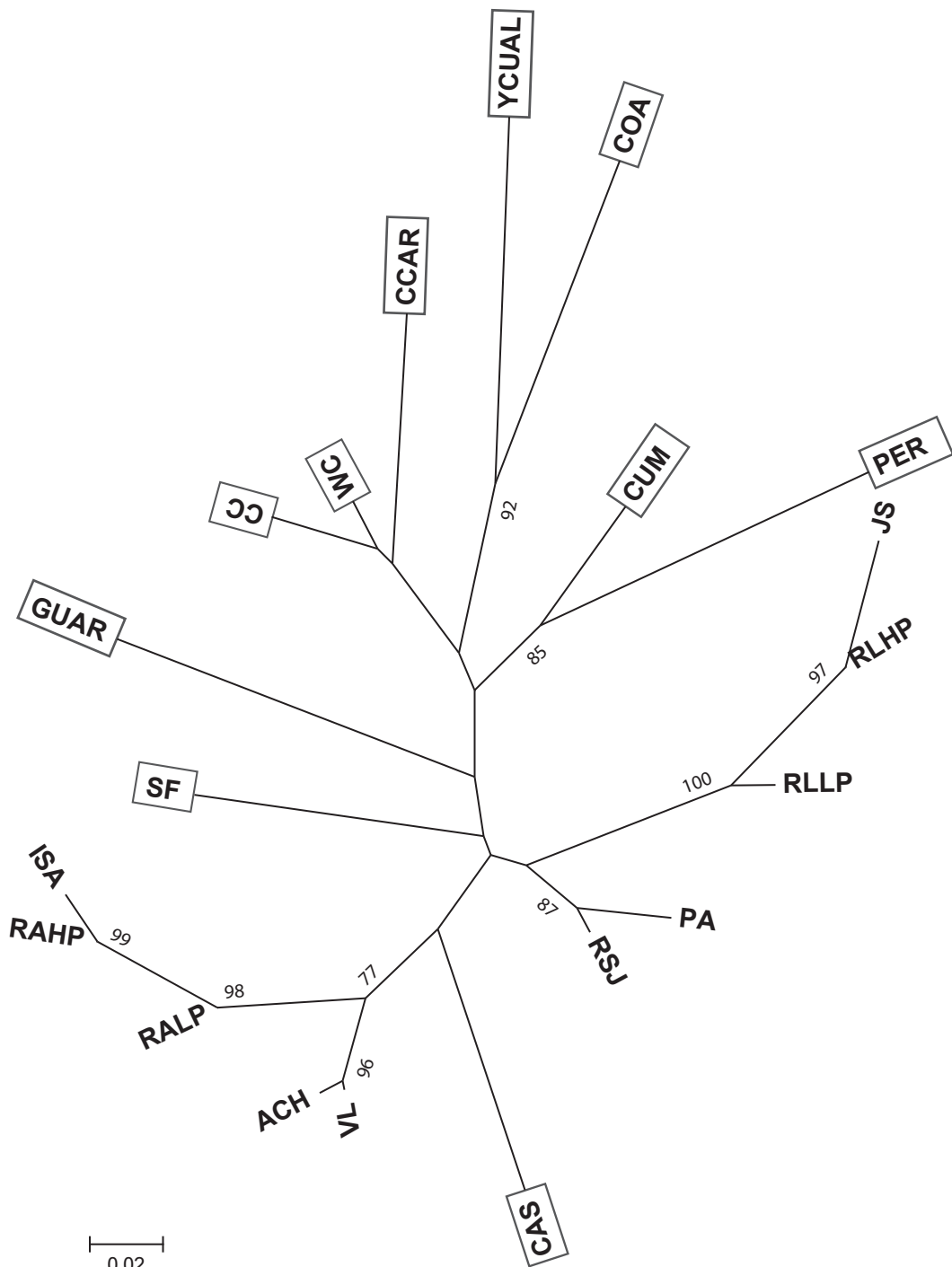

Supplement: Additional file 3: Figure S2 — Neighbor Joining tree showing relationships among populations. The tree was constructed from the matrix of pairwise FST and its robustness was tested with 1000 bootstrap replicates. Cariaco drainage populations are in frames. [file 1471-2148-14-28-S3.pdf]

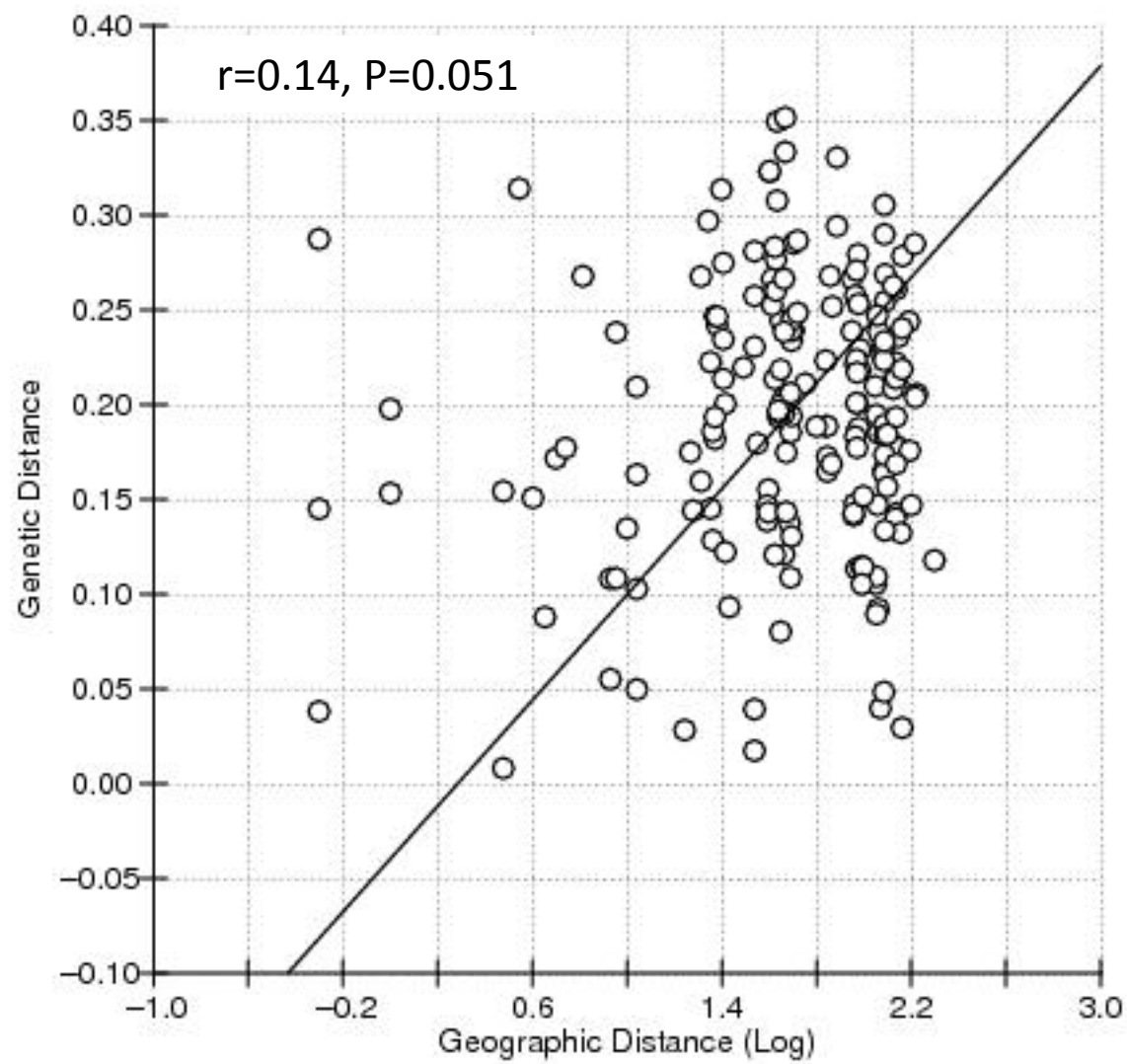

Supplement: Additional file 4: Figure S3 — Isolation by distance. Relationship between genetic (FST) and geographic distances between populations; r = 0.14, P = 0.051. [file 1471-2148-14-28-S4.pdf]

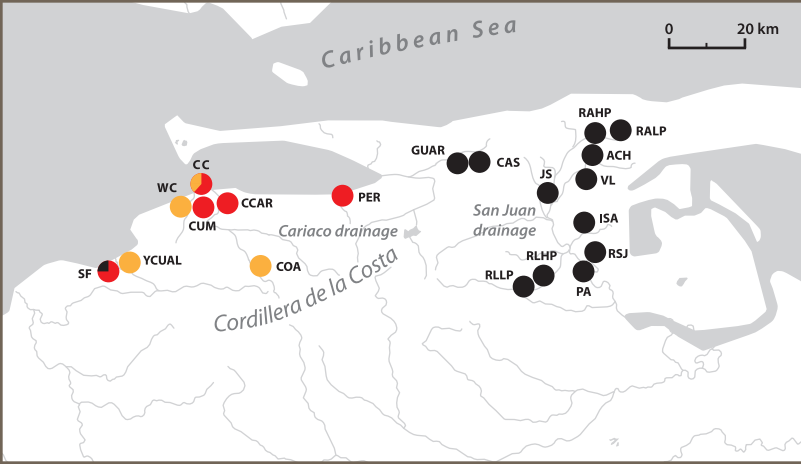

Supplement: Additional file 5: Figure S5. — Geographic distribution of mitochondrial lineages. Colours correspond to the three main lineages: A – black, B – yellow, C – red. [file 1471-2148-14-28-S5.pdf]

**Value of BIC  
versus number of clusters**

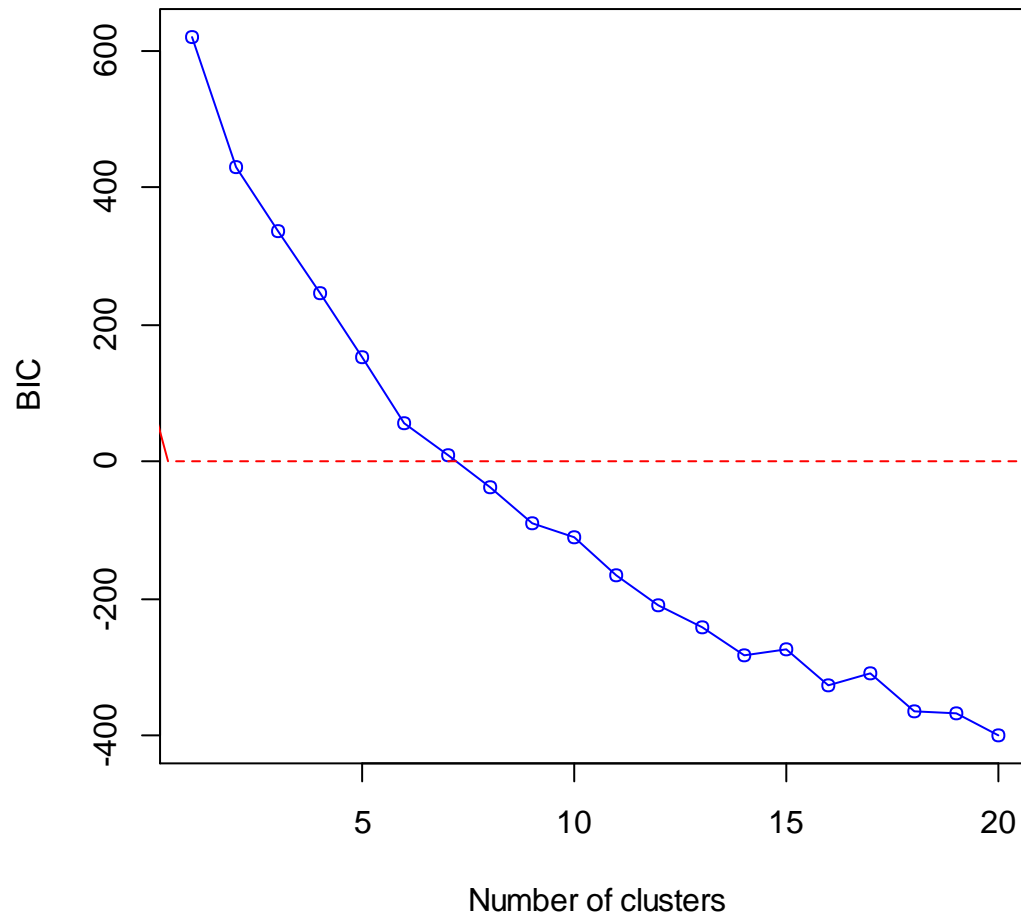

Supplement: Additional file 6: Figure S4 — Inference of the optimal number of genetic clusters. Graph output of the function find.clusters performed on morphometric data to identify the optimal number of clusters; Bayesian information criterion (BIC) on y axis. [file 1471-2148-14-28-S6.pdf]
